# 4-Nitro­phenyl 2-bromo-2-methyl­propano­ate

**DOI:** 10.1107/S1600536811005988

**Published:** 2011-02-23

**Authors:** Corrado Rizzoli, Elda Marku, Lucedio Greci

**Affiliations:** a Dipartimento di Chimica Generale ed Inorganica, Chimica Analitica, Chimica Fisica, Universitá degli Studi di Parma, Viale G. P. Usberti 17/A, I-43124 Parma, Italy; b Fakulteti i Shkencave të Natyrës, Departamenti i Kimise, Universiteti i Tiranes, Bulevardi "Zogu I", Tirana, Albania; c Dipartimento ISAC, Universitá Politecnica delle Marche, Via Brecce Bianche, I-60131 Ancona, Italy

## Abstract

In the title compound, C_10_H_10_BrNO_4_, the planes of the carboxyl­ate and nitro groups are rotated by 60.53 (13) and 6.4 (3)°, respectively, to the benzene ring. In the crystal, inter­molecular C—H⋯O hydrogen bonds link the mol­ecules into zigzag chains parallel to the *c* axis.

## Related literature

For the synthesis and biological properties of the title compound and analogues, see: Bischoff (1907 ▶); Kaeriyama *et al.* (1976 ▶). For the use of the title compound in organic synthesis, see: Haddleton & Waterson (1999 ▶); Edeleva *et al.* (2009 ▶); Guillaneuf *et al.* (2007 ▶).
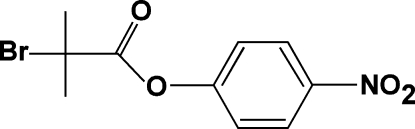


## Experimental

### 

#### Crystal data

C_10_H_10_BrNO_4_
*M* *_r_* = 288.10
Orthorhombic, 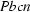

*a* = 11.4128 (16) Å
*b* = 14.450 (2) Å
*c* = 14.539 (2) Å
*V* = 2397.7 (6) Å^3^
*Z* = 8
Mo *K*α radiation
μ = 3.43 mm^−1^
*T* = 295 K
0.45 × 0.15 × 0.14 mm

#### Data collection

Bruker APEXII CCD diffractometer
Absorption correction: multi-scan (*SADABS*; Bruker, 2008 ▶) *T*_min_ = 0.625, *T*_max_ = 0.720
18926 measured reflections
2177 independent reflections
1086 reflections with *I* > 2σ(*I*)
*R*_int_ = 0.049

#### Refinement

*R*[*F* ^2^ > 2σ(*F* ^2^)] = 0.046
*wR*(*F* ^2^) = 0.132
*S* = 1.00
2177 reflections
145 parameters
H-atom parameters constrained
Δρ_max_ = 0.84 e Å^−3^
Δρ_min_ = −0.51 e Å^−3^

### 

Data collection: *APEX2* (Bruker, 2008 ▶); cell refinement: *SAINT* (Bruker, 2008 ▶); data reduction: *SAINT*; program(s) used to solve structure: *SIR97* (Altomare *et al.*, 1999 ▶); program(s) used to refine structure: *SHELXL97* (Sheldrick, 2008 ▶); molecular graphics: *ORTEP-3 for Windows* (Farrugia, 1997 ▶) and *SCHAKAL97* (Keller, 1997 ▶); software used to prepare material for publication: *SHELXL97* and *PARST95* (Nardelli, 1995 ▶).

## Supplementary Material

Crystal structure: contains datablocks global, I. DOI: 10.1107/S1600536811005988/tk2722sup1.cif

Structure factors: contains datablocks I. DOI: 10.1107/S1600536811005988/tk2722Isup2.hkl

Additional supplementary materials:  crystallographic information; 3D view; checkCIF report

## Figures and Tables

**Table 1:** Hydrogen-bond geometry (Å, °)

*D*—H⋯*A*	*D*—H	H⋯*A*	*D*⋯*A*	*D*—H⋯*A*
C6—H6⋯O3^i^	0.93	2.59	3.385 (4)	144

Symmetry code: (i) 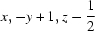.

**Fig. 1.**
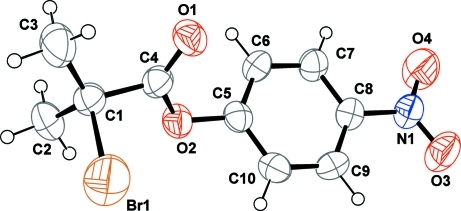
The molecular structure of the title compound, with displacement ellipsoids drawn at the 40% probability level.

**Fig. 2.**
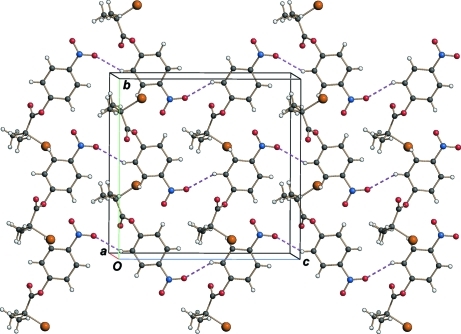
Partial crystal packing of the title compound viewed approximately along the a axis. Intermolecular C—H···O hydrogen bonds are shown as dashed lines.

## supplementary crystallographic information

### Comment

The synthesis of the title compound and analogues was originally reported in the early part of last century (Bischoff, 1907), and in the seventies several of these compounds were found to possess useful miticidal, insecticidal, nematocidal or fungicidal activities (Kaeriyama *et al.*, 1976). More recently, the title compound was prepared (Haddleton & Waterson, 1999) and used as initiator for H-atom transfer polymerization. The same compound was also used in the preparation of new alkoxyamines derived from imadaziline-, imidazoline- and pyrrolidine-1-oxyl nitroxides (Edeleva *et al.*, 2009) and, within our group, for the synthesis of phenyl- and 4-nitrophenyl- 2-(2,2-diphenyl-3-(phenylimino)-indolin-1-yloxy)-2-methylpropionate (Guillaneuf *et al.*, 2007). In order to obtain structural parameters for molecular mechanics calculations for the above mentioned alkoxyamines, the X-ray crystal structure of the title compound has been determined and the results are reported herein.

In the molecule of the title compound (Fig. 1), the plane of the nitro group is approximately coplanar with the benzene ring (dihedral angle 6.4 (3) °), whereas the plane of the carboxylic group is tilted by 60.53 (13) °. All bond lengths and angles are unexceptional. In the crystal structure (Fig. 2), the molecules are linked by intermolecular C—H···O hydrogen bonds (Table 1) into zigzag chains running parallel to the *c* axis.

### Experimental

The title compound was prepared according to the literature method (Haddleton & Waterson, 1999). Crystals suitable for X-ray analysis were obtained by slow evaporation of its *n*-pentane solution (m. p. 342–343 K). IR data, ν, cm^-1^: 1753 (C═O), 1615 and 1592 (benzene ring), 1521 (NO_2_). ^1^H NMR spectrum, δ in CDCl_3_: 2.08 (s, 6H); 7.3 (2*H*, d, *J* = 9.2 Hz); 8.31 (2*H*, d, *J* = 9.2 Hz). The ESI-MS obtained with 3200 QTRAP spectrometer does not give the molecular ion peak. The melting point was measured by an electrothermal apparatus and is uncorrected. The ^1^H NMR spectrum was recorded with a Varian 400 MHz spectrometer. The IR spectrum was recorded with a Perkin-Elmer MGX1 spectrophotometer.

### Refinement

All H atoms were placed in geometrically idealized positions and treated as riding atoms, with C—H = 0.93–0.96 Å, and *U*_iso_(H) = 1.2 *U*_eq_(C) or 1.5 *U*_eq_(C) for methyl H atoms.

### Crystal data

**Table:**

C_10_H_10_BrNO_4_	*F*(000) = 1152
*M**_r_* = 288.10	*D*_x_ = 1.596 Mg m^−^^3^
Orthorhombic, *P**b**c**n*	Mo *K*α radiation, λ = 0.71073 Å
Hall symbol: -P 2n 2ab	Cell parameters from 877 reflections
*a* = 11.4128 (16) Å	θ = 5.6–21.3°
*b* = 14.450 (2) Å	µ = 3.43 mm^−^^1^
*c* = 14.539 (2) Å	*T* = 295 K
*V* = 2397.7 (6) Å^3^	Needle, colourless
*Z* = 8	0.45 × 0.15 × 0.14 mm

### Data collection

**Table:**

Bruker APEXII CCD diffractometer	2177 independent reflections
Radiation source: fine-focus sealed tube	1086 reflections with *I* > 2σ(*I*)
graphite	*R*_int_ = 0.049
ω scans	θ_max_ = 25.3°, θ_min_ = 2.3°
Absorption correction: multi-scan (*SADABS*; Bruker, 2008)	*h* = −13→13
*T*_min_ = 0.625, *T*_max_ = 0.720	*k* = −15→17
18926 measured reflections	*l* = −17→17

### Refinement

**Table:**

Refinement on *F*^2^	Primary atom site location: structure-invariant direct methods
Least-squares matrix: full	Secondary atom site location: difference Fourier map
*R*[*F*^2^ > 2σ(*F*^2^)] = 0.046	Hydrogen site location: inferred from neighbouring sites
*wR*(*F*^2^) = 0.132	H-atom parameters constrained
*S* = 1.00	*w* = 1/[σ^2^(*F*_o_^2^) + (0.0685*P*)^2^] where *P* = (*F*_o_^2^ + 2*F*_c_^2^)/3
2177 reflections	(Δ/σ)_max_ < 0.001
145 parameters	Δρ_max_ = 0.84 e Å^−^^3^
0 restraints	Δρ_min_ = −0.51 e Å^−^^3^

### Special details

**Table:**

Refinement. Refinement of *F*^2^ against ALL reflections. The weighted *R*-factor *wR* and goodness of fit *S* are based on *F*^2^, conventional *R*-factors *R* are based on *F*, with *F* set to zero for negative *F*^2^. The threshold expression of *F*^2^ > σ(*F*^2^) is used only for calculating *R*-factors(gt) *etc*. and is not relevant to the choice of reflections for refinement. *R*-factors based on *F*^2^ are statistically about twice as large as those based on *F*, and *R*- factors based on ALL data will be even larger.

### Fractional atomic coordinates and isotropic or equivalent isotropic displacement parameters (Å^2^)

**Table:**

	*x*	*y*	*z*	*U*_iso_*/*U*_eq_	
Br1	−0.00330 (6)	0.10818 (4)	0.62160 (4)	0.1233 (3)	
O1	−0.0531 (2)	0.33291 (18)	0.53800 (18)	0.0757 (8)	
O2	0.1275 (2)	0.30016 (16)	0.58917 (16)	0.0639 (7)	
O3	0.1600 (3)	0.6175 (2)	0.8793 (2)	0.1042 (11)	
O4	0.1583 (4)	0.6983 (2)	0.7567 (2)	0.1188 (13)	
N1	0.1583 (3)	0.6243 (3)	0.7961 (3)	0.0724 (9)	
C1	0.0266 (3)	0.1820 (2)	0.5074 (2)	0.0603 (10)	
C2	0.1417 (3)	0.1512 (2)	0.4701 (3)	0.0783 (12)	
H2A	0.2016	0.1614	0.5154	0.117*	
H2B	0.1597	0.1859	0.4156	0.117*	
H2C	0.1380	0.0865	0.4554	0.117*	
C3	−0.0733 (4)	0.1656 (3)	0.4429 (4)	0.1049 (17)	
H3A	−0.1451	0.1849	0.4714	0.157*	
H3B	−0.0777	0.1009	0.4283	0.157*	
H3C	−0.0612	0.2005	0.3875	0.157*	
C4	0.0267 (3)	0.2799 (3)	0.5452 (2)	0.0603 (10)	
C5	0.1339 (3)	0.3821 (2)	0.6389 (3)	0.0551 (9)	
C6	0.1295 (3)	0.4663 (3)	0.5954 (2)	0.0628 (10)	
H6	0.1207	0.4695	0.5319	0.075*	
C8	0.1531 (3)	0.5392 (3)	0.7411 (2)	0.0556 (9)	
C7	0.1381 (3)	0.5461 (3)	0.6467 (3)	0.0626 (10)	
H7	0.1339	0.6038	0.6184	0.075*	
C9	0.1598 (3)	0.4549 (3)	0.7842 (2)	0.0618 (10)	
H9	0.1699	0.4515	0.8476	0.074*	
C10	0.1515 (3)	0.3756 (3)	0.7324 (3)	0.0623 (10)	
H10	0.1576	0.3179	0.7604	0.075*	

### Atomic displacement parameters (Å^2^)

**Table:**

	*U*^11^	*U*^22^	*U*^33^	*U*^12^	*U*^13^	*U*^23^
Br1	0.1760 (7)	0.0961 (5)	0.0979 (5)	−0.0278 (3)	0.0319 (3)	0.0105 (3)
O1	0.0642 (16)	0.0717 (18)	0.091 (2)	0.0147 (14)	−0.0123 (15)	−0.0147 (15)
O2	0.0549 (15)	0.0642 (17)	0.0727 (16)	0.0074 (13)	−0.0079 (13)	−0.0145 (14)
O3	0.153 (3)	0.095 (2)	0.064 (2)	−0.023 (2)	−0.0036 (18)	−0.0151 (16)
O4	0.198 (4)	0.061 (2)	0.097 (2)	−0.017 (2)	−0.004 (2)	−0.0031 (19)
N1	0.084 (2)	0.071 (3)	0.062 (2)	−0.0081 (19)	0.0088 (19)	−0.009 (2)
C1	0.058 (2)	0.062 (2)	0.061 (2)	0.0014 (18)	−0.0044 (18)	−0.0028 (19)
C2	0.080 (3)	0.062 (3)	0.093 (3)	0.014 (2)	0.012 (2)	−0.005 (2)
C3	0.097 (3)	0.087 (3)	0.131 (4)	0.018 (3)	−0.043 (3)	−0.047 (3)
C4	0.053 (2)	0.070 (3)	0.058 (2)	0.006 (2)	−0.0084 (19)	−0.007 (2)
C5	0.0414 (19)	0.060 (2)	0.064 (2)	0.0041 (17)	0.0010 (17)	−0.006 (2)
C6	0.069 (2)	0.075 (3)	0.045 (2)	−0.001 (2)	0.0076 (18)	−0.001 (2)
C8	0.046 (2)	0.064 (3)	0.056 (2)	−0.0086 (18)	0.0046 (16)	−0.007 (2)
C7	0.071 (2)	0.059 (3)	0.057 (2)	−0.003 (2)	0.0135 (19)	0.003 (2)
C9	0.062 (2)	0.073 (3)	0.050 (2)	−0.002 (2)	−0.0102 (18)	0.005 (2)
C10	0.058 (2)	0.055 (2)	0.074 (3)	0.0015 (19)	−0.0109 (19)	0.0033 (19)

### Geometric parameters (Å, °)

**Table:**

Br1—C1	2.002 (4)	C3—H3A	0.9600
O1—C4	1.195 (4)	C3—H3B	0.9600
O2—C4	1.348 (4)	C3—H3C	0.9600
O2—C5	1.390 (4)	C5—C6	1.372 (5)
O3—N1	1.214 (4)	C5—C10	1.378 (5)
O4—N1	1.214 (4)	C6—C7	1.376 (5)
N1—C8	1.467 (5)	C6—H6	0.9300
C1—C2	1.489 (5)	C8—C9	1.372 (5)
C1—C3	1.496 (5)	C8—C7	1.388 (5)
C1—C4	1.518 (5)	C7—H7	0.9300
C2—H2A	0.9600	C9—C10	1.375 (5)
C2—H2B	0.9600	C9—H9	0.9300
C2—H2C	0.9600	C10—H10	0.9300
			
C4—O2—C5	118.5 (3)	O1—C4—O2	123.6 (4)
O4—N1—O3	122.8 (4)	O1—C4—C1	124.4 (3)
O4—N1—C8	118.8 (3)	O2—C4—C1	112.0 (3)
O3—N1—C8	118.4 (4)	C6—C5—C10	121.4 (4)
C2—C1—C3	113.4 (3)	C6—C5—O2	121.0 (3)
C2—C1—C4	114.2 (3)	C10—C5—O2	117.6 (3)
C3—C1—C4	112.0 (3)	C5—C6—C7	119.4 (3)
C2—C1—Br1	107.1 (2)	C5—C6—H6	120.3
C3—C1—Br1	107.8 (3)	C7—C6—H6	120.3
C4—C1—Br1	101.3 (2)	C9—C8—C7	121.5 (3)
C1—C2—H2A	109.5	C9—C8—N1	119.5 (4)
C1—C2—H2B	109.5	C7—C8—N1	118.9 (3)
H2A—C2—H2B	109.5	C6—C7—C8	119.0 (4)
C1—C2—H2C	109.5	C6—C7—H7	120.5
H2A—C2—H2C	109.5	C8—C7—H7	120.5
H2B—C2—H2C	109.5	C8—C9—C10	119.1 (4)
C1—C3—H3A	109.5	C8—C9—H9	120.4
C1—C3—H3B	109.5	C10—C9—H9	120.4
H3A—C3—H3B	109.5	C9—C10—C5	119.5 (4)
C1—C3—H3C	109.5	C9—C10—H10	120.2
H3A—C3—H3C	109.5	C5—C10—H10	120.2
H3B—C3—H3C	109.5		

### Hydrogen-bond geometry (Å, °)

**Table:**

*D*—H···*A*	*D*—H	H···*A*	*D*···*A*	*D*—H···*A*
C6—H6···O3^i^	0.93	2.59	3.385 (4)	144

Symmetry codes: (i) *x*, −*y*+1, *z*−1/2.
